# Novel Microbial Diagnostic Methods for Clinical, Environmental, and Food Samples

**DOI:** 10.1155/2017/3942801

**Published:** 2017-07-31

**Authors:** Pratik Banerjee, Irshad M. Sulaiman, György Schneider, Udayan Ray, Bala Jagadeesan

**Affiliations:** ^1^Division of Epidemiology, Biostatistics, and Environmental Health Science, School of Public Health, University of Memphis, Memphis, TN, USA; ^2^Southeast Regional Laboratory, U.S. Food and Drug Administration, Atlanta, GA, USA; ^3^Department of Medical Microbiology and Immunology, University of Pécs, Pécs, Hungary; ^4^Royal Hobart Hospital, University of Tasmania School of Medicine, Hobart, TAS, Australia; ^5^Nestlé Research Center, Lausanne, Switzerland

The field of molecular diagnostics has experienced a remarkable growth and advancement in recent years resulting in significant improvement in disease diagnosis and intervention. Rapid, sensitive, and accurate detection and identification of microbial entities are critical issues for ensuring timeliness of clinical, environmental, and food safety interventions. With the advent of new technologies in the field of biomedical sciences, the resolution of microbial detection methods has improved tremendously. Currently, most of the advanced microbial detection methods involve multidisciplinary expertise (such as biology, chemistry, physics, engineering, material science, genomics, statistics, and bioinformatics). The advancement of rapid microbial diagnostics fueled by these disciplines along with the growth of closely associated fields like nanotechnology and “omics” has brought opportunities as well as an immense challenge for researchers and practitioners to adopt appropriate methods conducive to their respective applications and sample types.

The methods and approaches of microbial detection may vary quite significantly depending on the type of microbes and the nature of the sample or specimen under investigation. It is intuitive that “one size” does not fit all microbial diagnostics strategies. As a result, the proper choice of diagnostic modality is very important. Several new strategies of microbial detection have emerged in recent years with improved sensitivity, discriminatory power, and most importantly, a higher degree of accuracy. The clinical, environmental, and food samples often confer hostile environments to microbes. Therefore, the microorganisms inhabiting in these matrices may become injured or dead. In certain scenarios, microbial cells attain a viable but not culturable state in these matrices. Moreover, they may undergo additional stress during sample transport and subsequent preparations. All these factors may ultimately pose a significant challenge for proper microbial diagnosis if the detection methods are solely dependent on the ability of the target organisms to grow under laboratory or test conditions. Some molecular diagnostic techniques such as PCR and gene sequencing/metagenomics can be used in a culture independent manner and seems to address some of the long standing limitations (as described above) of microbial detection by growth-based (culture-dependent) methods.

This special issue provides a platform for sharing recent scientific advancements to researchers and practitioners working in the broader public health area, including clinical diagnostics, environmental scientists, and food scientists working in food safety and food microbiology area. The articles included in this special issue include both original scientific studies and review papers focused on microbial detection and characterization methods and application in clinical, food, agriculture, and environmental fields. Articles dealing with sample preparation, various human and animal disease diagnosis methods, gut microbiota, biotechnological application of microbes in food production, and environmental monitoring are included in this issue.

Appropriateness of sample preparation is one of the most essential prerequisites of efficient detection of microbial entities from complex sample matrices. Application of magnetic separation methods has emerged as a powerful technique for separation of biological entities (including intact cells or DNA) from clinical, food, or environmental samples. In their review article, M. Husakova et al. provide an overview of available information regarding* Mycobacterium avium* subsp.* paratuberculosis* (MAP) cells and DNA isolation methods based on magnetic separation procedures for the detection of MAP from a broad range of matrices including feces, blood, cheese, milk, and so on.

Several original research and review articles published in this issue highlight methods improvement as well as novel diagnostic method development. The potential use of* Treponema pallidum* recombinant antigens for diagnostic purposes was summarized in the review paper by A. Kubanov et al. The paper gives an overview of candidate surface-exposed proteins, adhesins, periplasmic, and flagellar proteins that could potentially be utilized for improved syphilis serological tests. Also, the authors highlight the fact that differentiation of early and late syphilis stages can be carried out by using these recombinant antigens. T. Matare et al. report an alternative method for testing the germ tube formation of* Candida* species. This approach is comparable to the one that uses the human serum for this purpose. Authors found that bicarbonate and trismaleate can induce germ tube formation. The optimal concentration of bicarbonate was found to be 20 mM at pH 5.5–6.5. The results also demonstrate that addition of 5–10% CO_2_ to the incubation atmosphere increased the rate of germ tube formation.

Optochin susceptibility is important parameters that differentiate between* Streptococcus pneumonia* and other streptococci. Although it is a frequently used method, a significant methodological discrepancy can be found in the literature due to the lack of a standardized method. By studying different factors (strain, agar, and serotype) I. Burckhardt et al. used statistical methods to evaluate an ideal reading time after inoculation, and their result suggests shortening the incubation time from 24 h to 12 h.

Rapid and sensitive detection of human and animal prion diseases is crucial to prevent potential morbidity and mortality inflicted by this pathogenic agent. The diagnostic modalities of prions are significantly different from other pathogenic microbes such as bacteria, viruses, and fungi owing to the differences in biological properties and mode of host-pathogen interactions. The review article by H.-E. Kang et al. summarizes aspects of prion biology and the assay principle, performance, and conditions of contemporary methods of diagnosis with particular emphasis on the real-time quaking-induced conversion (RT-QuIC) method in both human and animal specimens. In another paper, a protocol for evaluating the prevalence of multidrug resistance mutations in people with HIV-1 infection is suggested by S. Gupta et al. Their research focuses on patients with low viremia and targets the protease and reverse transcriptase regions of HIV-1. The well-optimized protocol is sensitive and reproducible for different HIV-1 subtypes and overcomes the limitations of narrow HIV subtype coverage.

Microbial community composition and diversity of a host or host-derived sample may be implicated with various outcomes including disease status, the spread of pathogens, fermentation profiles in foods, and so on. With the advent of high-throughput sequencing technologies, the resolution of microbial diagnostics has improved considerably. A vast majority of nonculturable microbes can be detected by Next Generation Sequencing (NGS) methods. Additionally, the NGS-metagenomics approach sheds light to shift of microbial community structure and may reveal changes in the diversity of the constituent microbiota at different taxonomic levels. This information may become important to understand disease pathology or even identify “biomarker” microbes. L. Cui et al. report the probable association of coronary heart disease (CHD) with an altered diversity and composition of gut microbiota. They evaluated the gut microbiome of CHD patients and compared that with healthy volunteers by using high-throughput sequencing of 16S ribosomal RNA genes. The results show that the diversity and composition of gut flora were different between CHD patients and healthy controls. For example, at phylum level Firmicutes and Fusobacteria were found to be higher in CHD patients compared with the controls. The results of this study suggest that an alteration of gut microbiota may be associated with the incidence of CHD.

Survival and persistence of pathogens in the environment may increase the risk of subsequent transmission to humans.* Ascaris lumbricoides* is a leading cause of helminthic diseases globally. The presence of parasitic helminth eggs is an indicator of sanitary risk and water quality. Therefore, rapid and sensitive detection of parasitic helminth egg is critical for risk assessment of water supply and sanitation. In their article, L. A. Soto et al. describe the applicability of two molecular techniques, quantitative PCR (qPCR) and digital PCR (dPCR), for detection of* A. lumbricoides* eggs in reclaimed water. The authors also report the development of two new DNA extraction protocols for the PCR assays. Their results reveal that both qPCR and dPCR can be utilized to detect DNA from* A. lumbricoides* eggs in reclaimed water with the limits of detection of conforming to the World Health Organization's recommended parameters.

In their paper, A. Abdelazez et al. examined different factors affecting survival and activity of* Bifidobacterium* species in the manufacturing of frozen yogurt under different conditions. They also evaluated the effect of storage temperatures on their viability in frozen yogurt, physicochemical, rheological, and the sensory parameters of the product. The results of this study show that the* Bifidobacterium* spp. can grow well in simulated intestinal conditions and can be used to manufacture frozen yogurt with this probiotic bacteria. They also demonstrate that the supplementation of* Bifidobacterium* has no adverse effect on the shelf life, flavor, or compositional characteristics of the frozen yogurt product.

Environmental parameters such as temperature may affect the growth, biological, and physiological activities of microorganisms and may also affect their detection. In their article, Y. Xu et al. reported the capability of a novel hypothermia aerobic nitrite-denitrifying bacterium,* Pseudomonas putida* strain Y-9 (a common inhabitant of long-term flooded paddy soil), to perform heterotrophic nitrification with ammonium and aerobic denitrification with nitrate or nitrite at low temperature (15°C). According to the results presented in this study, the denitrification ability of* P. putida* Y-9 may potentially be utilized for wastewater treatment to reduce nitrogen pollution under cold temperature conditions.

In summary, this editorial provides a snapshot of the articles published in this special issue. We hope the articles contained in this special issue will advance our knowledge and understanding of microbial detection field, and the readers of this special issue will find some information of interest to their respective area of scientific investigations.

